# What services are currently provided to people with heart failure with preserved ejection fraction in the UK, and what are their components? A protocol for a scoping literature review

**DOI:** 10.1093/eurjcn/zvae119

**Published:** 2024-08-26

**Authors:** Faye Forsyth, Christi Deaton, Paul R Kalra, Mark Green, Mary E Harrison, Sara Tavares, Andreas Dirksen, Isla Kuhn, Barbara Farquharson, Rosalynn C Austin

**Affiliations:** Department of Public Health and Primary Care, University of Cambridge, Robinson Way, Cambridge CB2 0SR, UK; KU Leuven Department of Public Health and Primary Care, KU Leuven—University of Leuven, Kapucijnenvoer 7, PB7001, Leuven 3000, Belgium; Department of Public Health and Primary Care, University of Cambridge, East Forvie Building, Cambridge CB2 0SR, UK; Department of Cardiology, Portsmouth Hospitals University NHS Trust, Portsmouth PO6 3LY, UK; Department of Cardiology, Portsmouth Hospitals University NHS Trust, Portsmouth PO6 3LY, UK; Leicester Diabetes Centre, University Hospitals of Leicester NHS Trust, Infirmary Square, Leicester LE1 5WW, UK; Department of Cardiovascular Sciences, University of Leicester, University Road, Leicester LE1 7RH, UK; Heart Failure Offices, Ealing Community Cardiology, Imperial College NHS Trust, Praed Street, London W2 1NY, UK; Med 1, Klinikum Darmstadt, Grafenstraße 9, Darmstadt 64285, Germany; Medical Library, University of Cambridge, Cambridge CB2 0QQ, UK; NMAHP Research Unit, Scion House, Stirling FK9 4NF, UK; Department of Cardiology, Portsmouth Hospitals University NHS Trust, Portsmouth PO6 3LY, UK; Department of Public Health, Faculty of Health Sciences, University of Stavanger, Stavanger 4021, Norway; NIHR Applied Research Collaborative (ARC) Wessex, Innovation Centre, Science Park, 2 Venture Rd, Chilworth, Southampton SO16 7NP, UK

**Keywords:** Clinical services, Heart failure, Heart failure with preserved ejection fraction, Scoping review

## Abstract

**Aims:**

Heart failure (HF) with preserved ejection fraction (HFpEF) is increasing in incidence and is increasingly the most common HF diagnosis. Patients with HFpEF are often excluded from specialist HF services, which has negative impacts on their healthcare experiences and health-related outcomes. As emerging evidence-based treatments are being incorporated into clinical guidelines, it is timely to focus on the management of this phenotype. This review aims to explore literature around care provision for HFpEF in the UK, to characterize and assess HFpEF care pathways against current standards, and to generate evidence to create an optimized framework of care.

**Methods and results:**

A scoping review of the evidence from six databases will be performed, alongside a search of grey literature search and consultation with relevant experts. Given the expected heterogeneity, multiple lines of synthesis are anticipated. Data analysis will follow best practice guidelines for the synthesizing methodologies selected. Patient and public representatives will assist with analysis and in identifying priority components for HFpEF clinical services.

**Conclusion:**

This scoping literature review will enable an in-depth examination of the current health service provision for those with HFpEF in the UK. Synthesis of key components of services and illumination of challenges and barriers will inform current and future practice. There is a long history of specialist HF care in the UK, including seminal work on nurse-led care. Therefore, evidence derived from this review will likely be useful to HF services across Europe. The proposed combination of the search across both peer-reviewed literature and grey literature, combined with patient and public involvement, will identify the key components of a framework of care for those with HFpEF.

**Registration:**

This scoping review protocol was published on the public Open Science Framework platform (no registration reference provided) and can be accessed at: https://osf.io/5gufq/.

## Graphical Abstract

**Graphical Abstract:**
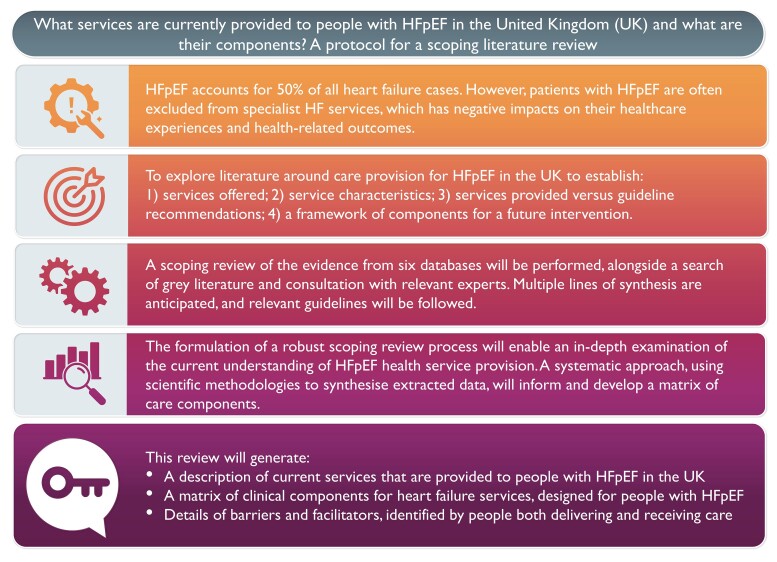

**Novelty**

- Recent advances in the pharmacological management of patients with heart failure with preserved ejection fraction necessitate a review of the organization of care.
- This review will characterize care being delivered and assess this against guideline recommendations.
- Summarizing care provision and its relative strengths/weaknesses will drive improvement in care.
- Given the established history of heart failure care in the UK, evidence derived from the review will be useful for, and transferable to, new or less well-established services across Europe.

## Introduction

Heart failure (HF) with preserved ejection fraction (HFpEF), defined as symptoms of HF within the context of a left ventricular ejection fraction ≥ 50%, structural remodelling, and/or diastolic dysfunction plus abnormal biomarkers,^1^ accounts for 50% of all HF cases and is growing in prevalence.^2–4^ Within the UK, care for people with HFpEF is usually embedded within services designed around the needs of people with HF with reduced ejection fraction (HFrEF).^5^ Recent advances in the pharmacological management of HFpEF^6,7^ has refocused attention on management and led some to question the suitability of current services.^3,8,9^

Much of the previous research exploring care provision in HFpEF has described barriers to optimum care.^5,10–12^ Key amongst these are as follows: low awareness of HFpEF,^5,13^ scepticism around the validity of HFpEF as a diagnosis^14,15^; physical barriers to diagnosis and management such as high natriuretic peptide levels^16^; funding restrictions that limit support from specialist services^17^; clinical inertia due to perceptions around treatability^18^; and uncertainties around responsibilities.^19^ For people living with HFpEF, these barriers can result in protracted diagnostic experiences and unsatisfactory levels of treatment and support.^10^ Whilst there is consensus amongst the HF community that change is needed,^20,21^ there is little literature characterizing and assessing ‘real-world’ HFpEF clinical services to help drive improvements.

### Rationale

The 2021 European Society of Cardiology guidelines state that those with HFpEF should receive multi-disciplinary team care that includes the management of co-morbidities, relief from congestion, support with self-management, and referral to cardiac rehabilitation.^1^ Similar recommendations are included in the National Institute for Health and Care Excellence^22^ guidelines in England and Scottish Intercollegiate Guidelines Network in Scotland.^23^ Recent research has demonstrated that novel pharmacological treatments can reduce symptoms and hospital admissions and improve quality of life in HFpEF.^24–26^ In response, guidelines have issued updates recommending the initiation of the sodium-glucose co-transporter-2 inhibitors (dapagliflozin and empagliflozin) in HFpEF.^27–29^

Previous research has shown that supportive care for those with HFpEF is not always available and is beset by challenges.^10,30,31^ Subsequently, patients with HFpEF are at risk of missing out on new pharmacological agents. There are few reports describing what services currently deliver in terms of care for people with HFpEF and even fewer describing how new therapies will be implemented within existing structures. Questions about capacity and capability to deliver additional care have already been raised.^32,33^ There is also emerging evidence that suggests expansion of HF services to include HFpEF may lead to dilution of care for people with HFrEF.^34^ Given these challenges, sharing information on effective organization and delivery of care is essential to improve outcomes. As the UK has a long history of delivering HF care and has led the way in delivering HF specialist nurse-driven care,^35,36^ information generated will likely be valuable and transferable to other settings. In summary, this scoping review will be the first to explore and synthesize what clinical services are available in the UK for people with HFpEF and to report and evaluate what features of care are being delivered.

### Objectives

The review has four aims: (i) to establish what services are currently offered to patients with HFpEF in the UK; (ii) to analyse and synthesize the characteristics of HFpEF services; (iii) to explore services provision against guideline recommendations; and (iv) to generate knowledge that would contribute to a framework that would guide a future clinical intervention.

### Patient and public involvement

A patient and public involvement and engagement (PPIE) group advised on this protocol and will continue to participate in this programme of work. Following synthesis, data will be circulated amongst the PPIE group, and they will be asked to contribute towards the interpretation of the data. Opportunities for more in-depth involvement will be offered throughout (i.e. as co-investigator.) The PPIE group will then be invited to evaluate the proposed new framework that is developed from the synthesis of the review.

## Methods

This report has been structured in line with the Preferred Reporting Items for Systematic reviews and Meta-Analyses extension for scoping reviews (PRISMA-ScR) checklist.^37^

### Protocol and registration

This scoping review protocol was published on the public Open Science Framework platform (no registration reference provided) and can be accessed at: https://osf.io/5gufq/.

### Eligibility criteria

We will consider publications of any type that (i) are published in the English language, (ii) describe or evaluate the content of HFpEF services within the UK, and (iii) have a publication date of ≥2013. A description of detailed inclusion principles based on Sample, Phenomenon of Interest, Design, Evaluation, Research type^38^ criteria is provided in *Table 1*.

**Table 1:** Sample, Phenomenon of Interest, Design, Evaluation, Research type criteria

Criterion	Description
Sample	People with diagnosed HFpEF seen in both community or hospital clinics. Heart failure services often treat all HF phenotypes; we will consider reports only when identification of HF phenotype is possible. Heart failure with preserved ejection fraction definitions and diagnostic criteria have rapidly evolved; therefore, non-explicit papers will be considered to determine presence of HFpEF. Mixed samples (i.e. HFpEF/HFrEF) will be included if there is sufficient detail parsing the phenotypes. No other exclusion criteria will be applied.
Phenomenon of Interest	Any healthcare service that provides supportive care to people with HFpEF with a focus on the management of their illness.
Design	Any publication that refers to ‘real-world’ management of HFpEF, including, but not limited to, retrospective case reviews, poster abstracts, retrospective observational studies, registry analyses, case reports, narrative descriptions, audits, and charitable organization reports.
Evaluation	Descriptions of the healthcare services provided will be examined for the following factors: type of services, referral criteria, services offered, follow-up schedules, patient-reported outcome measures (PROMs) collected, and staffing metrics.
Research type	All type of research articles together with grey literature will be included if published in English and since 2013.

### Information sources

Search terms were developed in conjunction with an information specialist (I.K.). Search terms for HFpEF will be based on previous reviews conducted by the authors.^39,40^ These terms reflected historical and continued debate over terminology related to HFpEF.^41^ We did not add search terms relating to clinical interactions (e.g. HF clinic, HF unit, and outpatient) as pilot searches demonstrated these negatively impacting search specificity and sensitivity.

Searches were performed in six bibliographic databases based on the recommendations from Bramer *et al.*^42^ [MEDLINE (via Ovid), Embase (via OVID), EMCARE (via Ovid), CINAHL (via EBSCO), Cochrane Library, and Web of Science]. Databases were searched separately, rather than multiple databases being searched on the same platform. The search syntax was adapted for each database and to account for variation between thesaurus terms and controlled vocabulary across each database. The UK filters have been adapted from Ayiku *et al*.^43,44^ Results were imported to Endnote by IK for de-duplication, using established methods.^45^ The search strategy was evaluated against the PRISMA-S guidelines.^46^ Searches included the period 1 January 2013 to 23 August 2023; to ensure only contemporary practice is included (prior to 2013, there was less consensus around the definition and diagnostic criteria of HFpEF). The searches will all be re-run prior to submission in order to include any papers published between the initial searching and submission for peer review.

Grey literature searches will be conducted in Google Scholar. The first 500 Google Scholar results will be screened and unique reports identified by consensus. Additionally, we will search information published by charities and National Health Service bodies responsible for commissioning, managing, or evaluating HF care. We anticipate data of interest may be published locally as ‘spotlights’, local pathways of care, cases, or internal reports (charitable/governmental). Appeals for HF specialists to share details on current clinical services that support those with HFpEF will be made through our individual networks, national HF networks, and social media. Backwards and forwards citation tracking will be performed.

### Search

Searches have been run and the full electronic searches are presented in the Supplementary material.

### Selection of sources of evidence

The digital platform Rayyan^47^ will be used to manage the screening of the search results. This will be performed blinded and in duplicate, by a team of researchers who have previous experience of conducting systematic, mixed methods, and scoping reviews, and meta-analysis. The team includes four HF specialist nurses and a practising cardiologist who bring the necessary expertise in assessing and evaluating care. In addition, team members come from a wide geographical area, which will facilitate access to data across a broad range of networks.

First-level screening will confirm eligibility based on title and abstract, possible inclusion of HFpEF in the sample, and whether clinical practice is or potentially described. Disagreements will be discussed between screeners and adjudicated by a third reviewer if necessary. Any publication included after first-level screening will undergo full-text review. Full-text review will also be performed blinded and in duplicate.

### Data charting process

Before charting begins, multiple reports relating to the same service will be aggregated, in line with best practice.^48^ The data extraction template will be piloted and modified as needed following extraction of the first five reports included in the review. Various tools will be employed to manage data extraction including Microsoft Excel^49^ and NVivo software.^50^ The charting process will be informed by established guidelines, including, but not limited to, Cochrane,^48^ Joanna Briggs Institute,^51^ and Systematic Review without Meta-Analysis (SWiM).^52^ Extraction will be performed independently by one researcher; a sub-section of extracted data will be checked by a second investigator. Authors of the original reports will be contacted to provide missing data or to clarify descriptive data if required.

### Data items

A broad range of data will be considered in this review. Data extraction fields and descriptions are detailed in *Table 2* below.

**Table 2:** Data extraction fields and descriptions

Data field	Description
Administrative	Title, author, acronym, year, country, design (main), design (sub-type), and sample details (if any) will be both summarized and considered for meta-analysis.
HFpEF population	Details of the HFpEF population (e.g. clinical characteristics) will be extracted if described, for example, age, gender, ejection fraction, other echocardiographic variables, comorbidities, socio-demographic factors, frailty data, and medication data.
Service characteristics	Staff, type of service (outpatient/inpatient), referrals, clinic sessions, follow-up, triage, tele-health, medication management, titration details, rehabilitation, and additional services. The TIDieR^53^ (template for intervention description and replication) framework will be used to organize and report service characteristics.
Results/findings	*Quantitative articles/reports*: primary outcome, secondary outcome, follow-up schedule, follow-up time point used in analysis, raw outcome data if any, and authors’ conclusions.
	*Qualitative articles/reports*: all published data along with the analysis of the data will be examined for descriptive details of the clinical encounter, details around the patient experience of the clinical interaction, met/unmet needs, clinician experiences and reflections, services offered, subsequent outcomes/referrals, etc. Of particular interest will be authors’ suggestions for the structure of a service aimed at this population. *Mixed-methods articles*: data from quantitative and qualitative data will be extracted and disaggregated accordingly.

### Critical appraisal of individual sources of evidence

Critical appraisal is not a requirement of scoping reviews; however, we consider this a necessary step that will inform the weighting we apply to results within the analysis. Study quality will be assessed using a relevant tool that corresponds to the reported study design. Given the wide variety of potential methods employed, we anticipate using the following: the Joanna Briggs Institute Critical Appraisal Skills Programme,^54^ Cochrane Risk of Bias assessment tool,^48^ Mixed Method Appraisal Tool (MMAT),^55^ and/or risk of bias in observational studies of interventions (ROBINS-I).^56^ Grey literature will most likely be evaluated using the Joanna Briggs Institute methodology of systematic reviews of text and opinion.^57^ However, each article retrieved will be evaluated on a case-by-case basis, depending on the data collected and analysis methods utilized.

### Synthesis of results

We expect this review will generate mixed data (qualitative and quantitative reports). Therefore, the synthesis will vary depending on the type and heterogeneity of the data. Guidelines that informed the charting process (Cochrane,^48^ Joanna Briggs Institute,^51^ and SWiM)^52^ will also be used to guide the synthesis methods. Qualitative synthesis and analysis will follow the principles of abductive analysis,^58^ as a means of theorizing reports to create a framework of components for supportive care in HFpEF. Quantitative data, if of sufficient quantity, will be assessed for suitability for meta-analysis.^48^ If, as is anticipated, there is insufficient data for meta-analysis, quantitative data will be reported in line with SWiM guidelines.^52^ Given the scope of the potential data we will retrieve, the exact synthesis methods will only be clear after extraction. Updated methods will be summarized in the online protocol hosted on the Open Science Framework platform^59^ and during the reporting of the review.

### Results of analysis

The formulation of robust search strategies will enable an in-depth examination of the current understanding of HFpEF health service provision in the UK. A systematic approach, using scientific methodologies to synthesize extracted data, will inform and develop a matrix of care components for those with HFpEF. This matrix will then be presented to PPIE groups and stakeholders for refinement, as a part of a larger piece of work to inform the development of a clinical intervention for those with HFpEF. The PRISMA-ScR will be utilized for reporting the review conduct.^37^ We expect to create various reports including a peer-reviewed publication and patient-focused outputs which we will co-design with the PPIE group. In addition, we anticipate sharing the matrix of components with the HF specialist nursing community, for their critical appraisal and revision.

## Discussion

Heart failure with preserved ejection fraction is a growing challenge that health services need to address. The adoption of new evidenced-based treatments for those with HFpEF is a welcome addition for clinicians who may often struggle to manage these individuals. This will be the first review to synthesize data enabling an initial description of current services provided to those with HFpEF. It will also be the first to provide a framework of clinical components for a HF service designed specifically for people with HFpEF. Further, it will collate barriers and facilitators, identified by people both delivering and receiving care. Together, these data will promote discussion around effective organization of care and provide services with the information they need to facilitate change that will deliver better outcomes for people with HFpEF.

## Conclusion

It is anticipated the results of this review will be beneficial for the informing of clinical services for those with HFpEF within the UK and beyond. Findings may inform the creation of supportive interventions and care pathways for this currently under severed population of patients.

## Supplementary Material

zvae119_Supplementary_Data

## Data Availability

Data, once available, will be made upon reasonable request.
