# Supplementary material for: What services are currently provided to people with heart failure with preserved ejection fraction in the UK, and what are their components? A protocol for a scoping literature review
Source: Eur J Cardiovasc Nurs. 2024 Aug 26;24(1):83–8. doi: 10.1093/eurjcn/zvae119 (PMC11781374; doi:10.1093/eurjcn/zvae119)
Supplement: zvae119_Supplementary_Data [file zvae119_supplementary_data.docx]

# Supplementary Material

Contents

[Supplementary Material 1](#_Toc155942523)

[Searches 2](#_Toc155942524)

[Search Results summary 2](#_Toc155942525)

[Medline 3](#_Toc155942526)

[Embase 5](#_Toc155942527)

[EMCARE 7](#_Toc155942528)

[CINAHL 9](#_Toc155942529)

[Cochrane 17](#_Toc155942530)

[Web of Science Core Collection 20](#_Toc155942531)

# Searches

The databases (Medline, Embase and Emcare via OVID, CINAHL via Ebsco, Cochrane Library, and Web of Science Core Collection) will be searched from 1^st^ January 2013 **to 23 August 2023** by IK

# Search Results summary

| MEDLINE ALL | 2560 |
| --- | --- |
| Embase | 5298 |
| Emcare | 1989 |
| CINAHL | 1666 |
| Cochrane | 658 |
| Web of Science Core Collection | 6915 |
| Total | 19,086 |
| No duplicates removed | 7639 |
| Total unique hits | 11447 |

# Medline

Ovid MEDLINE(R) ALL <1946 to August 22, 2023>

1 ("HFpEF" or "HFnEF" or "diastolic heart failure*" or "diastolic failure*" or "chronic heart failure*" or "ejection fraction*").ti,ab,kw,kf. or exp heart failure, diastolic/ 100068

2 ("ambulatory clinic" or "outpatient clinic*" or "virtual clinic*" or ((remote or home) adj2 monitor*) or "home care" or "home visit*" or "house call*" or "specialist nurse*" or "clinic*" or "multi-disciplinary team*" or "multidisciplinary team*" or MDT or "multi-disciplinary care" or "multidisciplinary care" or "nurse-led care" or "heart failure unit" or "heart function unit" or "outreach" or "disease management program*" or "community led care" or "community service*" or "community health service*" or "community clinic*" or "community health centre*" or "community health center*" or " pharmacy led care" or "pharmacy service*" or "pharmaceutic* service*" or "AHP led care" or "allied health professional led care" or "allied health personnel led care").ti,ab,kw,kf. 5353508

3 exp ambulatory care facilities/ or exp outpatient clinics, hospital/ or exp home care services/ or exp house calls/ or exp nurse specialists/ or exp patient care team/ or exp disease management/ or exp "delivery of health care"/ or exp community health services/ or exp community health centers/ or exp community pharmacy services/ 1604808

4 2 or 3 6571952

5 exp United Kingdom/ 390730

6 (national health service* or nhs*).ti,ab,in. 273070

7 (english not ((published or publication* or translat* or written or language* or speak* or literature or citation*) adj5 english)).ti,ab. 50126

8 (gb or "g.b." or britain* or (british* not "british columbia") or uk or "u.k." or united kingdom* or (england* not "new england") or northern ireland* or northern irish* or scotland* or scottish* or ((wales or "south wales") not "new south wales") or welsh*).ti,ab,jw,in. 2460817

9 (bath or "bath's" or ((birmingham not alabama*) or ("birmingham's" not alabama*) or bradford or "bradford's" or brighton or "brighton's" or bristol or "bristol's" or carlisle* or "carlisle's" or (cambridge not (massachusetts* or boston* or harvard*)) or ("cambridge's" not (massachusetts* or boston* or harvard*)) or (canterbury not zealand*) or ("canterbury's" not zealand*) or chelmsford or "chelmsford's" or chester or "chester's" or chichester or "chichester's" or coventry or "coventry's" or derby or "derby's" or (durham not (carolina* or nc)) or ("durham's" not (carolina* or nc)) or ely or "ely's" or exeter or "exeter's" or gloucester or "gloucester's" or hereford or "hereford's" or hull or "hull's" or lancaster or "lancaster's" or leeds* or leicester or "leicester's" or (lincoln not nebraska*) or ("lincoln's" not nebraska*) or (liverpool not (new south wales* or nsw)) or ("liverpool's" not (new south wales* or nsw)) or ((london not (ontario* or ont or toronto*)) or ("london's" not (ontario* or ont or toronto*)) or manchester or "manchester's" or (newcastle not (new south wales* or nsw)) or ("newcastle's" not (new south wales* or nsw)) or norwich or "norwich's" or nottingham or "nottingham's" or oxford or "oxford's" or peterborough or "peterborough's" or plymouth or "plymouth's" or portsmouth or "portsmouth's" or preston or "preston's" or ripon or "ripon's" or salford or "salford's" or salisbury or "salisbury's" or sheffield or "sheffield's" or southampton or "southampton's" or st albans or stoke or "stoke's" or sunderland or "sunderland's" or truro or "truro's" or wakefield or "wakefield's" or wells or westminster or "westminster's" or winchester or "winchester's" or wolverhampton or "wolverhampton's" or (worcester not (massachusetts* or boston* or harvard*)) or ("worcester's" not (massachusetts* or boston* or harvard*)) or (york not ("new york*" or ny or ontario* or ont or toronto*)) or ("york's" not ("new york*" or ny or ontario* or ont or toronto*))))).ti,ab,in. 1756803

10 (bangor or "bangor's" or cardiff or "cardiff's" or newport or "newport's" or st asaph or "st asaph's" or st davids or swansea or "swansea's").ti,ab,in. 70786

11 (aberdeen or "aberdeen's" or dundee or "dundee's" or edinburgh or "edinburgh's" or glasgow or "glasgow's" or inverness or (perth not australia*) or ("perth's" not australia*) or stirling or "stirling's").ti,ab,in. 258729

12 (armagh or "armagh's" or belfast or "belfast's" or lisburn or "lisburn's" or londonderry or "londonderry's" or derry or "derry's" or newry or "newry's").ti,ab,in. 34063

13 or/5-12 3088273

14 (exp africa/ or exp americas/ or exp antarctic regions/ or exp arctic regions/ or exp asia/ or exp australia/ or exp oceania/) not (exp United Kingdom/ or europe/) 3340515

15 13 not 14 2922975

16 1 and 4 and 15 3661

17 limit 16 to yr="2013 -Current" 2560

# Embase

Embase <1974 to 2023 August 22>

1 ("HFpEF" or "HFnEF" or "diastolic heart failure*" or "diastolic failure*" or "chronic heart failure*" or "ejection fraction*").ti,ab. 186846

2 exp *diastolic heart failure/ 8147

3 1 or 2 187244

4 ("ambulatory clinic" or "outpatient clinic*" or "virtual clinic*" or ((remote or home) adj2 monitor*) or "home care" or "home visit*" or "house call*" or "specialist nurse*" or "clinic*" or "multi-disciplinary team*" or "multidisciplinary team*" or MDT or "multi-disciplinary care" or "multidisciplinary care" or "nurse-led care" or "heart failure unit" or "heart function unit" or "outreach" or "disease management program*" or "community led care" or "community service*" or "community health service*" or "community clinic*" or "community health centre*" or "community health center*" or " pharmacy led care" or "pharmacy service*" or "pharmaceutic* service*" or "AHP led care" or "allied health professional led care" or "allied health personnel led care").ti,ab. 7559696

5 exp *ambulatory care/ or exp *outpatient department/ or exp *home care/ or exp *home visit/ or exp *nurse specialist/ or exp *patient care team/ or exp *multidisciplinary team/ or exp *disease management/ or exp *health care delivery/ or exp *community care/ or exp *community health center/ 1382416

6 4 or 5 8468097

7 exp United Kingdom/ 463562

8 (national health service* or nhs*).ti,ab,in,ad. 468954

9 (english not ((published or publication* or translat* or written or language* or speak* or literature or citation*) adj5 english)).ti,ab. 60212

10 (gb or "g.b." or britain* or (british* not "british columbia") or uk or "u.k." or united kingdom* or (england* not "new england") or northern ireland* or northern irish* or scotland* or scottish* or ((wales or "south wales") not "new south wales") or welsh*).ti,ab,jx,in,ad. 3715245

11 (bath or "bath's" or ((birmingham not alabama*) or ("birmingham's" not alabama*) or bradford or "bradford's" or brighton or "brighton's" or bristol or "bristol's" or carlisle* or "carlisle's" or (cambridge not (massachusetts* or boston* or harvard*)) or ("cambridge's" not (massachusetts* or boston* or harvard*)) or (canterbury not zealand*) or ("canterbury's" not zealand*) or chelmsford or "chelmsford's" or chester or "chester's" or chichester or "chichester's" or coventry or "coventry's" or derby or "derby's" or (durham not (carolina* or nc)) or ("durham's" not (carolina* or nc)) or ely or "ely's" or exeter or "exeter's" or gloucester or "gloucester's" or hereford or "hereford's" or hull or "hull's" or lancaster or "lancaster's" or leeds* or leicester or "leicester's" or (lincoln not nebraska*) or ("lincoln's" not nebraska*) or (liverpool not (new south wales* or nsw)) or ("liverpool's" not (new south wales* or nsw)) or ((london not (ontario* or ont or toronto*)) or ("london's" not (ontario* or ont or toronto*)) or manchester or "manchester's" or (newcastle not (new south wales* or nsw)) or ("newcastle's" not (new south wales* or nsw)) or norwich or "norwich's" or nottingham or "nottingham's" or oxford or "oxford's" or peterborough or "peterborough's" or plymouth or "plymouth's" or portsmouth or "portsmouth's" or preston or "preston's" or ripon or "ripon's" or salford or "salford's" or salisbury or "salisbury's" or sheffield or "sheffield's" or southampton or "southampton's" or st albans or stoke or "stoke's" or sunderland or "sunderland's" or truro or "truro's" or wakefield or "wakefield's" or wells or westminster or "westminster's" or winchester or "winchester's" or wolverhampton or "wolverhampton's" or (worcester not (massachusetts* or boston* or harvard*)) or ("worcester's" not (massachusetts* or boston* or harvard*)) or (york not ("new york*" or ny or ontario* or ont or toronto*)) or ("york's" not ("new york*" or ny or ontario* or ont or toronto*))))).ti,ab,in,ad. 2904813

12 (bangor or "bangor's" or cardiff or "cardiff's" or newport or "newport's" or st asaph or "st asaph's" or st davids or swansea or "swansea's").ti,ab,in,ad. 119531

13 (aberdeen or "aberdeen's" or dundee or "dundee's" or edinburgh or "edinburgh's" or glasgow or "glasgow's" or inverness or (perth not australia*) or ("perth's" not australia*) or stirling or "stirling's").ti,ab,in,ad. 399958

14 (armagh or "armagh's" or belfast or "belfast's" or lisburn or "lisburn's" or londonderry or "londonderry's" or derry or "derry's" or newry or "newry's").ti,ab,in,ad. 55590

15 or/7-14 4540842

16 (exp "arctic and antarctic"/ or exp oceanic regions/ or exp western hemisphere/ or exp africa/ or exp asia/) not (exp united kingdom/ or europe/) 3514731

17 15 not 16 4284924

18 3 and 6 and 17 7690

19 limit 18 to yr="2013 -Current" 5298

# EMCARE

Ovid Emcare <1995 to 2023 Week 33>

1 ("HFpEF" or "HFnEF" or "diastolic heart failure*" or "diastolic failure*" or "chronic heart failure*" or "ejection fraction*").ti,ab. 35868

2 exp heart ejection fraction/ or exp heart failure with preserved ejection fraction/ 29258

3 exp diastolic heart failure/ 1350

4 1 or 2 or 3 47674

5 ("ambulatory clinic" or "outpatient clinic*" or "virtual clinic*" or ((remote or home) adj2 monitor*) or "home care" or "home visit*" or "house call*" or "specialist nurse*" or "clinic*" or "multi-disciplinary team*" or "multidisciplinary team*" or MDT or "multi-disciplinary care" or "multidisciplinary care" or "nurse-led care" or "heart failure unit" or "heart function unit" or "outreach" or "disease management program*" or "community led care" or "community service*" or "community health service*" or "community clinic*" or "community health centre*" or "community health center*" or " pharmacy led care" or "pharmacy service*" or "pharmaceutic* service*" or "AHP led care" or "allied health professional led care" or "allied health personnel led care").ti,ab. 1778757

6 exp ambulatory care/ or exp outpatient/ or exp home care/ or exp home visit/ or nurse specialist/ or exp patient care/ or exp disease management/ or exp health care delivery/ or exp community care/ or exp health center/ or exp "pharmacy (shop)"/ 1586532

7 5 or 6 2862812

8 exp United Kingdom/ 122433

9 (national health service* or nhs*).ti,ab,in,ad. 177814

10 (english not ((published or publication* or translat* or written or language* or speak* or literature or citation*) adj5 english)).ti,ab. 28430

11 (gb or "g.b." or britain* or (british* not "british columbia") or uk or "u.k." or united kingdom* or (england* not "new england") or northern ireland* or northern irish* or scotland* or scottish* or ((wales or "south wales") not "new south wales") or welsh*).ti,ab,jx,in,ad. 997957

12 (bath or "bath's" or ((birmingham not alabama*) or ("birmingham's" not alabama*) or bradford or "bradford's" or brighton or "brighton's" or bristol or "bristol's" or carlisle* or "carlisle's" or (cambridge not (massachusetts* or boston* or harvard*)) or ("cambridge's" not (massachusetts* or boston* or harvard*)) or (canterbury not zealand*) or ("canterbury's" not zealand*) or chelmsford or "chelmsford's" or chester or "chester's" or chichester or "chichester's" or coventry or "coventry's" or derby or "derby's" or (durham not (carolina* or nc)) or ("durham's" not (carolina* or nc)) or ely or "ely's" or exeter or "exeter's" or gloucester or "gloucester's" or hereford or "hereford's" or hull or "hull's" or lancaster or "lancaster's" or leeds* or leicester or "leicester's" or (lincoln not nebraska*) or ("lincoln's" not nebraska*) or (liverpool not (new south wales* or nsw)) or ("liverpool's" not (new south wales* or nsw)) or ((london not (ontario* or ont or toronto*)) or ("london's" not (ontario* or ont or toronto*)) or manchester or "manchester's" or (newcastle not (new south wales* or nsw)) or ("newcastle's" not (new south wales* or nsw)) or norwich or "norwich's" or nottingham or "nottingham's" or oxford or "oxford's" or peterborough or "peterborough's" or plymouth or "plymouth's" or portsmouth or "portsmouth's" or preston or "preston's" or ripon or "ripon's" or salford or "salford's" or salisbury or "salisbury's" or sheffield or "sheffield's" or southampton or "southampton's" or st albans or stoke or "stoke's" or sunderland or "sunderland's" or truro or "truro's" or wakefield or "wakefield's" or wells or westminster or "westminster's" or winchester or "winchester's" or wolverhampton or "wolverhampton's" or (worcester not (massachusetts* or boston* or harvard*)) or ("worcester's" not (massachusetts* or boston* or harvard*)) or (york not ("new york*" or ny or ontario* or ont or toronto*)) or ("york's" not ("new york*" or ny or ontario* or ont or toronto*))))).ti,ab,in,ad. 794653

13 (bangor or "bangor's" or cardiff or "cardiff's" or newport or "newport's" or st asaph or "st asaph's" or st davids or swansea or "swansea's").ti,ab,in,ad. 37574

14 (aberdeen or "aberdeen's" or dundee or "dundee's" or edinburgh or "edinburgh's" or glasgow or "glasgow's" or inverness or (perth not australia*) or ("perth's" not australia*) or stirling or "stirling's").ti,ab,in,ad. 105052

15 (armagh or "armagh's" or belfast or "belfast's" or lisburn or "lisburn's" or londonderry or "londonderry's" or derry or "derry's" or newry or "newry's").ti,ab,in,ad. 15966

16 or/8-15 1206898

17 4 and 7 and 16 3534

18 limit 17 to yr="2013 -Current" 1989

# CINAHL

| **#** | **Query** | **Limiters/Expanders** | **Last Run Via** | **Results** |
| --- | --- | --- | --- | --- |
| S1 | TI ( ("HFpEF" or "HFnEF" or "diastolic heart failure*" or "diastolic failure*" or "chronic heart failure*" or "ejection fraction*") ) OR AB ( ("HFpEF" or "HFnEF" or "diastolic heart failure*" or "diastolic failure*" or "chronic heart failure*" or "ejection fraction*") ) | Expanders - Apply equivalent subjects  Search modes - Boolean/Phrase | Interface - EBSCOhost Research Databases  Search Screen - Advanced Search  Database - CINAHL | 27,973 |
| S2 | TI ( ("ambulatory clinic" or "outpatient clinic*" or "virtual clinic*" or ((remote or home) n2 monitor*) or "home care" or "home visit*" or "house call*" or "specialist nurse*" or "clinic*" or "multi-disciplinary team*" or "multidisciplinary team*" or MDT or "multi-disciplinary care" or "multidisciplinary care" or "nurse-led care" or "heart failure unit" or "heart function unit" or "outreach" or "disease management program*" or "community led care" or "community service*" or "community health service*" or "community clinic*" or "community health centre*" or "community health center*" or " pharmacy led care" or "pharmacy service*" or "pharmaceutic* service*" or "AHP led care" or "allied health professional led care" or "allied health personnel led care") ) OR AB ( ("ambulatory clinic" or "outpatient clinic*" or "virtual clinic*" or ((remote or home) n2 monitor*) or "home care" or "home visit*" or "house call*" or "specialist nurse*" or "clinic*" or "multi-disciplinary team*" or "multidisciplinary team*" or MDT or "multi-disciplinary care" or "multidisciplinary care" or "nurse-led care" or "heart failure unit" or "heart function unit" or "outreach" or "disease management program*" or "community led care" or "community service*" or "community health service*" or "community clinic*" or "community health centre*" or "community health center*" or " pharmacy led care" or "pharmacy service*" or "pharmaceutic* service*" or "AHP led care" or "allied health professional led care" or "allied health personnel led care") ) | Expanders - Apply equivalent subjects  Search modes - Boolean/Phrase | Interface - EBSCOhost Research Databases  Search Screen - Advanced Search  Database - CINAHL | 1,376,701 |
| S3 | (MH "Ambulatory Care") OR (MH "Ambulatory Care Facilities+") OR (MH "Outpatient Service") OR (MH "Outpatients") OR (MH "Home Health Care+") OR (MH "Community Health Centers+") OR (MH "Hospitals, Community") OR (MH "Home Visits") OR (MH "Home Nursing") OR (MH "Nurse Specialist Service (Saba CCC)") OR (MH "Clinical Nurse Specialists+") OR (MH "Multidisciplinary Care Team+") OR (MH "Patient Care Plans+") OR (MH "Disease Management+") OR (MH "Health Care Delivery+") OR (MH "Community Health Services+") OR (MH "Pharmacy Service+") | Expanders - Apply equivalent subjects  Search modes - Boolean/Phrase | Interface - EBSCOhost Research Databases  Search Screen - Advanced Search  Database - CINAHL | 959,412 |
| S4 | S2 OR S3 | Expanders - Apply equivalent subjects  Search modes - Boolean/Phrase | Interface - EBSCOhost Research Databases  Search Screen - Advanced Search  Database - CINAHL | 2,129,789 |
| S5 | (MH "United Kingdom+") | Expanders - Apply equivalent subjects  Search modes - Boolean/Phrase | Interface - EBSCOhost Research Databases  Search Screen - Advanced Search  Database - CINAHL | 321,509 |
| S6 | TI ( (english not ((published or publication* or translat* or written or language* or speak* or literature or citation*) N5 english) ) OR AB ( (english not ((published or publication* or translat* or written or language* or speak* or literature or citation*) N5 english) ) | Expanders - Apply equivalent subjects  Search modes - Boolean/Phrase | Interface - EBSCOhost Research Databases  Search Screen - Advanced Search  Database - CINAHL | 20,947 |
| S7 | TI ( (gb or "g.b." or britain* or (british* not "british columbia") or uk or "u.k." or united kingdom* or (england* not "new england") or northern ireland* or northern irish* or scotland* or scottish* or ((wales or "south wales") not "new south wales") or welsh*) ) OR AB ( (gb or "g.b." or britain* or (british* not "british columbia") or uk or "u.k." or united kingdom* or (england* not "new england") or northern ireland* or northern irish* or scotland* or scottish* or ((wales or "south wales") not "new south wales") or welsh*) ) OR AF ( (gb or "g.b." or britain* or (british* not "british columbia") or uk or "u.k." or united kingdom* or (england* not "new england") or northern ireland* or northern irish* or scotland* or scottish* or ((wales or "south wales") not "new south wales") or welsh*) ) | Expanders - Apply equivalent subjects  Search modes - Boolean/Phrase | Interface - EBSCOhost Research Databases  Search Screen - Advanced Search  Database - CINAHL | 587,844 |
| S8 | TI ( (bath or "bath's" or ((birmingham not alabama*) or ("birmingham's" not alabama*) or bradford or "bradford's" or brighton or "brighton's" or bristol or "bristol's" or carlisle* or "carlisle's" or (cambridge not (massachusetts* or boston* or harvard*)) or ("cambridge's" not (massachusetts* or boston* or harvard*)) or (canterbury not zealand*) or ("canterbury's" not zealand*) or chelmsford or "chelmsford's" or chester or "chester's" or chichester or "chichester's" or coventry or "coventry's" or derby or "derby's" or (durham not (carolina* or nc)) or ("durham's" not (carolina* or nc)) or ely or "ely's" or exeter or "exeter's" or gloucester or "gloucester's" or hereford or "hereford's" or hull or "hull's" or lancaster or "lancaster's" or leeds* or leicester or "leicester's" or (lincoln not nebraska*) or ("lincoln's" not nebraska*) or (liverpool not (new south wales* or nsw)) or ("liverpool's" not (new south wales* or nsw)) or ((london not (ontario* or ont or toronto*)) or ("london's" not (ontario* or ont or toronto*)) or manchester or "manchester's" or (newcastle not (new south wales* or nsw)) or ("newcastle's" not (new south wales* or nsw)) or norwich or "norwich's" or nottingham or "nottingham's" or oxford or "oxford's" or peterborough or "peterborough's" or plymouth or "plymouth's" or portsmouth or "portsmouth's" or preston or "preston's" or ripon or "ripon's" or salford or "salford's" or salisbury or "salisbury's" or sheffield or "sheffield's" or southampton or "southampton's" or st albans or stoke or "stoke's" or sunderland or "sunderland's" or truro or "truro's" or wakefield or "wakefield's" or wells or westminster or "westminster's" or winchester or "winchester's" or wolverhampton or "wolverhampton's" or (worcester not (massachusetts* or boston* or harvard*)) or ("worcester's" not (massachusetts* or boston* or harvard*)) or (york not ("new york*" or ny or ontario* or ont or toronto*)) or ("york's" not ("new york*" or ny or ontario* or ont or toronto*))))) ) OR AB ( (bath or "bath's" or ((birmingham not alabama*) or ("birmingham's" not alabama*) or bradford or "bradford's" or brighton or "brighton's" or bristol or "bristol's" or carlisle* or "carlisle's" or (cambridge not (massachusetts* or boston* or harvard*)) or ("cambridge's" not (massachusetts* or boston* or harvard*)) or (canterbury not zealand*) or ("canterbury's" not zealand*) or chelmsford or "chelmsford's" or chester or "chester's" or chichester or "chichester's" or coventry or "coventry's" or derby or "derby's" or (durham not (carolina* or nc)) or ("durham's" not (carolina* or nc)) or ely or "ely's" or exeter or "exeter's" or gloucester or "gloucester's" or hereford or "hereford's" or hull or "hull's" or lancaster or "lancaster's" or leeds* or leicester or "leicester's" or (lincoln not nebraska*) or ("lincoln's" not nebraska*) or (liverpool not (new south wales* or nsw)) or ("liverpool's" not (new south wales* or nsw)) or ((london not (ontario* or ont or toronto*)) or ("london's" not (ontario* or ont or toronto*)) or manchester or "manchester's" or (newcastle not (new south wales* or nsw)) or ("newcastle's" not (new south wales* or nsw)) or norwich or "norwich's" or nottingham or "nottingham's" or oxford or "oxford's" or peterborough or "peterborough's" or plymouth or "plymouth's" or portsmouth or "portsmouth's" or preston or "preston's" or ripon or "ripon's" or salford or "salford's" or salisbury or "salisbury's" or sheffield or "sheffield's" or southampton or "southampton's" or st albans or stoke or "stoke's" or sunderland or "sunderland's" or truro or "truro's" or wakefield or "wakefield's" or wells or westminster or "westminster's" or winchester or "winchester's" or wolverhampton or "wolverhampton's" or (worcester not (massachusetts* or boston* or harvard*)) or ("worcester's" not (massachusetts* or boston* or harvard*)) or (york not ("new york*" or ny or ontario* or ont or toronto*)) or ("york's" not ("new york*" or ny or ontario* or ont or toronto*))))) ) OR AF ( (bath or "bath's" or ((birmingham not alabama*) or ("birmingham's" not alabama*) or bradford or "bradford's" or brighton or "brighton's" or bristol or "bristol's" or carlisle* or "carlisle's" or (cambridge not (massachusetts* or boston* or harvard*)) or ("cambridge's" not (massachusetts* or boston* or harvard*)) or (canterbury not zealand*) or ("canterbury's" not zealand*) or chelmsford or "chelmsford's" or chester or "chester's" or chichester or "chichester's" or coventry or "coventry's" or derby or "derby's" or (durham not (carolina* or nc)) or ("durham's" not (carolina* or nc)) or ely or "ely's" or exeter or "exeter's" or gloucester or "gloucester's" or hereford or "hereford's" or hull or "hull's" or lancaster or "lancaster's" or leeds* or leicester or "leicester's" or (lincoln not nebraska*) or ("lincoln's" not nebraska*) or (liverpool not (new south wales* or nsw)) or ("liverpool's" not (new south wales* or nsw)) or ((london not (ontario* or ont or toronto*)) or ("london's" not (ontario* or ont or toronto*)) or manchester or "manchester's" or (newcastle not (new south wales* or nsw)) or ("newcastle's" not (new south wales* or nsw)) or norwich or "norwich's" or nottingham or "nottingham's" or oxford or "oxford's" or peterborough or "peterborough's" or plymouth or "plymouth's" or portsmouth or "portsmouth's" or preston or "preston's" or ripon or "ripon's" or salford or "salford's" or salisbury or "salisbury's" or sheffield or "sheffield's" or southampton or "southampton's" or st albans or stoke or "stoke's" or sunderland or "sunderland's" or truro or "truro's" or wakefield or "wakefield's" or wells or westminster or "westminster's" or winchester or "winchester's" or wolverhampton or "wolverhampton's" or (worcester not (massachusetts* or boston* or harvard*)) or ("worcester's" not (massachusetts* or boston* or harvard*)) or (york not ("new york*" or ny or ontario* or ont or toronto*)) or ("york's" not ("new york*" or ny or ontario* or ont or toronto*))))) ) | Expanders - Apply equivalent subjects  Search modes - Boolean/Phrase | Interface - EBSCOhost Research Databases  Search Screen - Advanced Search  Database - CINAHL | 1,099,245 |
| S9 | TI ( (bangor or "bangor's" or cardiff or "cardiff's" or newport or "newport's" or st asaph or "st asaph's" or st davids or swansea or "swansea's") ) OR AB ( (bangor or "bangor's" or cardiff or "cardiff's" or newport or "newport's" or st asaph or "st asaph's" or st davids or swansea or "swansea's") ) OR AF ( (bangor or "bangor's" or cardiff or "cardiff's" or newport or "newport's" or st asaph or "st asaph's" or st davids or swansea or "swansea's") ) | Expanders - Apply equivalent subjects  Search modes - Boolean/Phrase | Interface - EBSCOhost Research Databases  Search Screen - Advanced Search  Database - CINAHL | 25,972 |
| S10 | TI ( (aberdeen or "aberdeen's" or dundee or "dundee's" or edinburgh or "edinburgh's" or glasgow or "glasgow's" or inverness or (perth not australia*) or ("perth's" not australia*) or stirling or "stirling's" ) OR AB ( (aberdeen or "aberdeen's" or dundee or "dundee's" or edinburgh or "edinburgh's" or glasgow or "glasgow's" or inverness or (perth not australia*) or ("perth's" not australia*) or stirling or "stirling's" ) OR AF ( (aberdeen or "aberdeen's" or dundee or "dundee's" or edinburgh or "edinburgh's" or glasgow or "glasgow's" or inverness or (perth not australia*) or ("perth's" not australia*) or stirling or "stirling's" ) | Expanders - Apply equivalent subjects  Search modes - Boolean/Phrase | Interface - EBSCOhost Research Databases  Search Screen - Advanced Search  Database - CINAHL | 71,138 |
| S11 | TI ( (armagh or "armagh's" or belfast or "belfast's" or lisburn or "lisburn's" or londonderry or "londonderry's" or derry or "derry's" or newry or "newry's") ) OR AB ( (armagh or "armagh's" or belfast or "belfast's" or lisburn or "lisburn's" or londonderry or "londonderry's" or derry or "derry's" or newry or "newry's") ) OR AF ( (armagh or "armagh's" or belfast or "belfast's" or lisburn or "lisburn's" or londonderry or "londonderry's" or derry or "derry's" or newry or "newry's") ) | Expanders - Apply equivalent subjects  Search modes - Boolean/Phrase | Interface - EBSCOhost Research Databases  Search Screen - Advanced Search  Database - CINAHL | 11,263 |
| S12 | S5 OR S6 OR S7 OR S8 OR S9 OR S10 OR S11 | Expanders - Apply equivalent subjects  Search modes - Boolean/Phrase | Interface - EBSCOhost Research Databases  Search Screen - Advanced Search  Database - CINAHL | 1,471,025 |
| S13 | (MH "Africa+") or (MH "America+") or (MH "Antarctic Regions") or (MH "North America+") OR (MH "South America+") or (MH "Arctic Regions") or (MH "Asia+") | Expanders - Apply equivalent subjects  Search modes - Boolean/Phrase | Interface - EBSCOhost Research Databases  Search Screen - Advanced Search  Database - CINAHL | 1,370,252 |
| S14 | (MH "Europe") OR (MH "United Kingdom+") | Expanders - Apply equivalent subjects  Search modes - Boolean/Phrase | Interface - EBSCOhost Research Databases  Search Screen - Advanced Search  Database - CINAHL | 352,737 |
| S15 | s13 not s14 | Expanders - Apply equivalent subjects  Search modes - Boolean/Phrase | Interface - EBSCOhost Research Databases  Search Screen - Advanced Search  Database - CINAHL | 1,347,580 |
| S16 | s12 not s15 | Expanders - Apply equivalent subjects  Search modes - Boolean/Phrase | Interface - EBSCOhost Research Databases  Search Screen - Advanced Search  Database - CINAHL | 1,310,822 |
| S17 | S1 AND S4 AND S16 | Expanders - Apply equivalent subjects  Search modes - Boolean/Phrase | Interface - EBSCOhost Research Databases  Search Screen - Advanced Search  Database - CINAHL | 2,458 |
| S18 | S1 AND S4 AND S16 | Limiters - Published Date: 20130101-20231231  Expanders - Apply equivalent subjects  Search modes - Boolean/Phrase | Interface - EBSCOhost Research Databases  Search Screen - Advanced Search  Database - CINAHL | 1,666 |

# Cochrane

Search Name:

Date Run: 23/08/2023 15:23:30

Comment:

ID Search Hits

#1 ((HFpEF or HFnEF or diastolic heart failure* or diastolic failure* or chronic heart failure* or ejection fraction*)):ti,ab,kw (Word variations have been searched) 29550

#2 MeSH descriptor: [Heart Failure, Diastolic] explode all trees 139

#3 #1 or #2 29550

#4 ((ambulatory clinic or (outpatient next clinic*) or (virtual next clinic*) or ((remote or home) near monitor*) or home care or (home next visit*) or (house next call*) or (specialist next nurse*) or clinic* or (multi-disciplinary next team*) or (multidisciplinary next team*) or MDT or multi-disciplinary care or multidisciplinary care or nurse-led care or heart failure unit or heart function unit or outreach or (disease next management next program*) or community led care or (community next service*) or (community next health next service*) or (community next clinic*) or (community next health next centre*) or (community next health next center*) or pharmacy led care or (pharmacy next service*) or (pharmaceutic* next service*) or AHP led care or allied health professional led care or allied health personnel led care)):ti,ab,kw (Word variations have been searched) 1059059

#5 MeSH descriptor: [Ambulatory Care] explode all trees 4131

#6 MeSH descriptor: [Ambulatory Care Facilities] explode all trees 2223

#7 MeSH descriptor: [] explode all trees 0

#8 MeSH descriptor: [Outpatient Clinics, Hospital] explode all trees 706

#9 MeSH descriptor: [Home Care Services, Hospital-Based] explode all trees 268

#10 MeSH descriptor: [House Calls] explode all trees 686

#11 MeSH descriptor: [Nurse Specialists] explode all trees 393

#12 MeSH descriptor: [Patient Care Team] explode all trees 2057

#13 MeSH descriptor: [Disease Management] explode all trees 6491

#14 MeSH descriptor: [Delivery of Health Care] explode all trees 61264

#15 MeSH descriptor: [Community Health Services] explode all trees 18508

#16 MeSH descriptor: [Community Health Centers] explode all trees 670

#17 MeSH descriptor: [Community Pharmacy Services] explode all trees 333

#18 #4 or #5 or #6 or #7 or #8 or #9 or #10 or #11 or #12 or #13 or #14 or #15 or #16 or #17 1086199

#19 MeSH descriptor: [United Kingdom] explode all trees 9391

#20 national health service* or nhs*:ti,ab,kw 16271

#21 (english not ((published or publication* or translat* or written or language* or speak* or literature or citation*) near/5 english)):ti,ab,kw 4452

#22 (gb or "g.b." or britain* or (british* not "british columbia") or uk or "u.k." or united kingdom* or (england* not "new england") or northern ireland* or northern irish* or scotland* or scottish* or ((wales or "south wales") not "new south wales") or welsh*):ti,ab,kw 39747

#23 (gb or "g.b." or britain* or (british* not "british columbia") or uk or "u.k." or united kingdom* or (england* not "new england") or northern ireland* or northern irish* or scotland* or scottish* or ((wales or "south wales") not "new south wales") or welsh*):so 71787

#24 (bath or "bath's" or ((birmingham not alabama*) or ("birmingham's" not alabama*) or bradford or "bradford's" or brighton or "brighton's" or bristol or "bristol's" or carlisle* or "carlisle's" or (cambridge not (massachusetts* or boston* or harvard*)) or ("cambridge's" not (massachusetts* or boston* or harvard*)) or (canterbury not zealand*) or ("canterbury's" not zealand*) or chelmsford or "chelmsford's" or chester or "chester's" or chichester or "chichester's" or coventry or "coventry's" or derby or "derby's" or (durham not (carolina* or nc)) or ("durham's" not (carolina* or nc)) or ely or "ely's" or exeter or "exeter's" or gloucester or "gloucester's" or hereford or "hereford's" or hull or "hull's" or lancaster or "lancaster's" or leeds* or leicester or "leicester's" or (lincoln not nebraska*) or ("lincoln's" not nebraska*) or (liverpool not (new south wales* or nsw)) or ("liverpool's" not (new south wales* or nsw)) or ((london not (ontario* or ont or toronto*)) or ("london's" not (ontario* or ont or toronto*)) or manchester or "manchester's" or (newcastle not (new south wales* or nsw)) or ("newcastle's" not (new south wales* or nsw)) or norwich or "norwich's" or nottingham or "nottingham's" or oxford or "oxford's" or peterborough or "peterborough's" or plymouth or "plymouth's" or portsmouth or "portsmouth's" or preston or "preston's" or ripon or "ripon's" or salford or "salford's" or salisbury or "salisbury's" or sheffield or "sheffield's" or southampton or "southampton's" or st albans or stoke or "stoke's" or sunderland or "sunderland's" or truro or "truro's" or wakefield or "wakefield's" or wells or westminster or "westminster's" or winchester or "winchester's" or wolverhampton or "wolverhampton's" or (worcester not (massachusetts* or boston* or harvard*)) or ("worcester's" not (massachusetts* or boston* or harvard*)) or (york not ("new york*" or ny or ontario* or ont or toronto*)) or ("york's" not ("new york*" or ny or ontario* or ont or toronto*))))):ti,ab,kw 24868

#25 (bangor or "bangor's" or cardiff or "cardiff's" or newport or "newport's" or st asaph or "st asaph's" or st davids or swansea or "swansea's"):ti,ab,kw 492

#26 (aberdeen or "aberdeen's" or dundee or "dundee's" or edinburgh or "edinburgh's" or glasgow or "glasgow's" or inverness or (perth not australia*) or ("perth's" not australia*) or stirling or "stirling's"):ti,ab,kw 7734

#27 armagh or "armagh's" or belfast or "belfast's" or lisburn or "lisburn's" or londonderry or "londonderry's" or derry or "derry's" or newry or "newry's":ti,ab,kw 1897

#28 #19 or #20 or #21 or #22 or #23 or #24 or #25 or #26 or #27 144276

#29 MeSH descriptor: [Africa] explode all trees 11466

#30 MeSH descriptor: [Americas] explode all trees 38004

#31 MeSH descriptor: [Antarctic Regions] explode all trees 15

#32 MeSH descriptor: [Asia] explode all trees 33436

#33 MeSH descriptor: [Oceania] explode all trees 7751

#34 #29 or #30 or #31 or #32 or #33 88162

#35 #28 not #34 137818

#36 #3 and #18 and #35 with Publication Year from 2013 to 2023, with Cochrane Library publication date Between Jan 2013 and Dec 2023, in Trials 658

# Web of Science Core Collection

# Web of Science Search Strategy (v0.1)

# Database: Web of Science Core Collection

# Entitlements:

- WOS.IC: 1993 to 2023

- WOS.CCR: 1985 to 2023

- WOS.SCI: 1900 to 2023

- WOS.AHCI: 1975 to 2023

- WOS.BHCI: 2008 to 2023

- WOS.BSCI: 2008 to 2023

- WOS.ESCI: 2018 to 2023

- WOS.ISTP: 1990 to 2023

- WOS.SSCI: 1956 to 2023

- WOS.ISSHP: 1990 to 2023

# Searches:

1: TS=(("HFpEF" or "HFnEF" or "diastolic heart failure*" or "diastolic failure*" or "chronic heart failure*" or "ejection fraction*")) Date Run: Wed Aug 23 2023 15:27:00 GMT+0100 (British Summer Time) Results: 115674

2: TS=("ambulatory clinic" or "outpatient clinic*" or "virtual clinic*" or ((remote or home) near/2 monitor*) or "home care" or "home visit*" or "house call*" or "specialist nurse*" or "clinic*" or "multi-disciplinary team*" or "multidisciplinary team*" or MDT or "multi-disciplinary care" or "multidisciplinary care" or "nurse-led care" or "heart failure unit" or "heart function unit" or "outreach" or "disease management program*" or "community led care" or "community service*" or "community health service*" or "community clinic*" or "community health centre*" or "community health center*" or " pharmacy led care" or "pharmacy service*" or "pharmaceutic* service*" or "AHP led care" or "allied health professional led care" or "allied health personnel led care") Date Run: Wed Aug 23 2023 15:27:20 GMT+0100 (British Summer Time) Results: 5076661

3: TS=((national health service* or nhs*)) or OO=(national health service* or nhs*) or AD=(national health service* or nhs*) Date Run: Wed Aug 23 2023 15:27:45 GMT+0100 (British Summer Time) Results: 396156

4: TS=((gb or "g.b." or britain* or (british* not "british columbia") or uk or "u.k." or “united kingdom*” or (england* not "new england") or “northern ireland*” or “northern irish*” or scotland* or scottish* or ((wales or "south wales") not "new south wales") or welsh*)) or OO=(gb or "g.b." or britain* or (british* not "british columbia") or uk or "u.k." or “united kingdom*” or (england* not "new england") or “northern ireland*” or “northern irish*” or scotland* or scottish* or ((wales or "south wales") not "new south wales") or welsh*) or AD=(gb or "g.b." or britain* or (british* not "british columbia") or uk or "u.k." or “united kingdom*” or (england* not "new england") or “northern ireland*” or “northern irish*” or scotland* or scottish* or ((wales or "south wales") not "new south wales") or welsh*) Date Run: Wed Aug 23 2023 15:28:11 GMT+0100 (British Summer Time) Results: 6756906

5: TS=((bath or "bath's" or ((birmingham not alabama*) or ("birmingham's" not alabama*) or bradford or "bradford's" or brighton or "brighton's" or bristol or "bristol's" or carlisle* or "carlisle's" or (cambridge not (massachusetts* or boston* or harvard*)) or ("cambridge's" not (massachusetts* or boston* or harvard*)) or (canterbury not zealand*) or ("canterbury's" not zealand*) or chelmsford or "chelmsford's" or chester or "chester's" or chichester or "chichester's" or coventry or "coventry's" or derby or "derby's" or (durham not (carolina* or nc)) or ("durham's" not (carolina* or nc)) or ely or "ely's" or exeter or "exeter's" or gloucester or "gloucester's" or hereford or "hereford's" or hull or "hull's" or lancaster or "lancaster's" or leeds* or leicester or "leicester's" or (lincoln not nebraska*) or ("lincoln's" not nebraska*) or (liverpool not (new south wales* or nsw)) or ("liverpool's" not (new south wales* or nsw)) or ((london not (ontario* or ont or toronto*)) or ("london's" not (ontario* or ont or toronto*)) or manchester or "manchester's" or (newcastle not (new south wales* or nsw)) or ("newcastle's" not (new south wales* or nsw)) or norwich or "norwich's" or nottingham or "nottingham's" or oxford or "oxford's" or peterborough or "peterborough's" or plymouth or "plymouth's" or portsmouth or "portsmouth's" or preston or "preston's" or ripon or "ripon's" or salford or "salford's" or salisbury or "salisbury's" or sheffield or "sheffield's" or southampton or "southampton's" or st albans or stoke or "stoke's" or sunderland or "sunderland's" or truro or "truro's" or wakefield or "wakefield's" or wells or westminster or "westminster's" or winchester or "winchester's" or wolverhampton or "wolverhampton's" or (worcester not (massachusetts* or boston* or harvard*)) or ("worcester's" not (massachusetts* or boston* or harvard*)) or (york not ("new york*" or ny or ontario* or ont or toronto*)) or ("york's" not ("new york*" or ny or ontario* or ont or toronto*)))))) or OO=(bath or "bath's" or ((birmingham not alabama*) or ("birmingham's" not alabama*) or bradford or "bradford's" or brighton or "brighton's" or bristol or "bristol's" or carlisle* or "carlisle's" or (cambridge not (massachusetts* or boston* or harvard*)) or ("cambridge's" not (massachusetts* or boston* or harvard*)) or (canterbury not zealand*) or ("canterbury's" not zealand*) or chelmsford or "chelmsford's" or chester or "chester's" or chichester or "chichester's" or coventry or "coventry's" or derby or "derby's" or (durham not (carolina* or nc)) or ("durham's" not (carolina* or nc)) or ely or "ely's" or exeter or "exeter's" or gloucester or "gloucester's" or hereford or "hereford's" or hull or "hull's" or lancaster or "lancaster's" or leeds* or leicester or "leicester's" or (lincoln not nebraska*) or ("lincoln's" not nebraska*) or (liverpool not (new south wales* or nsw)) or ("liverpool's" not (new south wales* or nsw)) or ((london not (ontario* or ont or toronto*)) or ("london's" not (ontario* or ont or toronto*)) or manchester or "manchester's" or (newcastle not (new south wales* or nsw)) or ("newcastle's" not (new south wales* or nsw)) or norwich or "norwich's" or nottingham or "nottingham's" or oxford or "oxford's" or peterborough or "peterborough's" or plymouth or "plymouth's" or portsmouth or "portsmouth's" or preston or "preston's" or ripon or "ripon's" or salford or "salford's" or salisbury or "salisbury's" or sheffield or "sheffield's" or southampton or "southampton's" or st albans or stoke or "stoke's" or sunderland or "sunderland's" or truro or "truro's" or wakefield or "wakefield's" or wells or westminster or "westminster's" or winchester or "winchester's" or wolverhampton or "wolverhampton's" or (worcester not (massachusetts* or boston* or harvard*)) or ("worcester's" not (massachusetts* or boston* or harvard*)) or (york not ("new york*" or ny or ontario* or ont or toronto*)) or ("york's" not ("new york*" or ny or ontario* or ont or toronto*))))) or AD=(bath or "bath's" or ((birmingham not alabama*) or ("birmingham's" not alabama*) or bradford or "bradford's" or brighton or "brighton's" or bristol or "bristol's" or carlisle* or "carlisle's" or (cambridge not (massachusetts* or boston* or harvard*)) or ("cambridge's" not (massachusetts* or boston* or harvard*)) or (canterbury not zealand*) or ("canterbury's" not zealand*) or chelmsford or "chelmsford's" or chester or "chester's" or chichester or "chichester's" or coventry or "coventry's" or derby or "derby's" or (durham not (carolina* or nc)) or ("durham's" not (carolina* or nc)) or ely or "ely's" or exeter or "exeter's" or gloucester or "gloucester's" or hereford or "hereford's" or hull or "hull's" or lancaster or "lancaster's" or leeds* or leicester or "leicester's" or (lincoln not nebraska*) or ("lincoln's" not nebraska*) or (liverpool not (new south wales* or nsw)) or ("liverpool's" not (new south wales* or nsw)) or ((london not (ontario* or ont or toronto*)) or ("london's" not (ontario* or ont or toronto*)) or manchester or "manchester's" or (newcastle not (new south wales* or nsw)) or ("newcastle's" not (new south wales* or nsw)) or norwich or "norwich's" or nottingham or "nottingham's" or oxford or "oxford's" or peterborough or "peterborough's" or plymouth or "plymouth's" or portsmouth or "portsmouth's" or preston or "preston's" or ripon or "ripon's" or salford or "salford's" or salisbury or "salisbury's" or sheffield or "sheffield's" or southampton or "southampton's" or st albans or stoke or "stoke's" or sunderland or "sunderland's" or truro or "truro's" or wakefield or "wakefield's" or wells or westminster or "westminster's" or winchester or "winchester's" or wolverhampton or "wolverhampton's" or (worcester not (massachusetts* or boston* or harvard*)) or ("worcester's" not (massachusetts* or boston* or harvard*)) or (york not ("new york*" or ny or ontario* or ont or toronto*)) or ("york's" not ("new york*" or ny or ontario* or ont or toronto*))))) Date Run: Wed Aug 23 2023 15:29:10 GMT+0100 (British Summer Time) Results: 12467264

6: TS=(bangor or "bangor's" or cardiff or "cardiff's" or newport or "newport's" or “st asaph” or "st asaph's" or “st davids” or swansea or "swansea's") or OO=(bangor or "bangor's" or cardiff or "cardiff's" or newport or "newport's" or “st asaph” or "st asaph's" or “st davids” or swansea or "swansea's") or AD=(bangor or "bangor's" or cardiff or "cardiff's" or newport or "newport's" or “st asaph” or "st asaph's" or “st davids” or swansea or "swansea's") Date Run: Wed Aug 23 2023 15:29:39 GMT+0100 (British Summer Time) Results: 248278

7: TS=(aberdeen or "aberdeen's" or dundee or "dundee's" or edinburgh or "edinburgh's" or glasgow or "glasgow's" or inverness or (perth not australia*) or ("perth's" not australia*) or stirling or "stirling's") or OO=(aberdeen or "aberdeen's" or dundee or "dundee's" or edinburgh or "edinburgh's" or glasgow or "glasgow's" or inverness or (perth not australia*) or ("perth's" not australia*) or stirling or "stirling's") or AD=(aberdeen or "aberdeen's" or dundee or "dundee's" or edinburgh or "edinburgh's" or glasgow or "glasgow's" or inverness or (perth not australia*) or ("perth's" not australia*) or stirling or "stirling's") Date Run: Wed Aug 23 2023 15:29:55 GMT+0100 (British Summer Time) Results: 775056

8: TS=(armagh or "armagh's" or belfast or "belfast's" or lisburn or "lisburn's" or londonderry or "londonderry's" or derry or "derry's" or newry or "newry's") or OO=(armagh or "armagh's" or belfast or "belfast's" or lisburn or "lisburn's" or londonderry or "londonderry's" or derry or "derry's" or newry or "newry's") or AD=(armagh or "armagh's" or belfast or "belfast's" or lisburn or "lisburn's" or londonderry or "londonderry's" or derry or "derry's" or newry or "newry's") Date Run: Wed Aug 23 2023 15:30:13 GMT+0100 (British Summer Time) Results: 121088

9: #3 OR #4 OR #5 OR #6 OR #7 OR #8 Date Run: Wed Aug 23 2023 15:30:34 GMT+0100 (British Summer Time) Results: 14572003

10: #9 AND #2 AND #1 Date Run: Wed Aug 23 2023 15:30:44 GMT+0100 (British Summer Time) Results: 10780

11: #9 AND #2 AND #1 Timespan: 2013-01-01 to 2023-12-31 Date Run: Wed Aug 23 2023 15:31:12 GMT+0100 (British Summer Time) Results: 6915
